# Group A Streptococcus: a case series of infective compartment syndrome

**DOI:** 10.1093/jscr/rjag610

**Published:** 2026-07-21

**Authors:** Augustin Msellati, Muireann Keating, Roisin Baker, Edward Jason Kelly

**Affiliations:** Department of Plastic Surgery, Cork University Hospital, Wilton, Cork, Co. Cork, T12 DC4A, Ireland; Department of Plastic Surgery, Cork University Hospital, Wilton, Cork, Co. Cork, T12 DC4A, Ireland; School of Medicine, Royal College of Surgeons in Ireland, 123 St Stephen's Green, Dublin, D02 YN77, Ireland; Department of Plastic Surgery, Cork University Hospital, Wilton, Cork, Co. Cork, T12 DC4A, Ireland; Department of Plastic Surgery, Cork University Hospital, Wilton, Cork, Co. Cork, T12 DC4A, Ireland

**Keywords:** Group A Streptococcus, compartment syndrome, upper limb, reconstruction, fasciotomy

## Abstract

Acute compartment syndrome develops when pressure within a closed osteofascial compartment rises above arterial pressure, disrupting local circulation. It is a surgical emergency; if untreated, it causes ischaemia and ultimately tissue necrosis. The majority of cases arise in the lower limbs secondary to fractures, although crush injuries, vascular injuries, burns, and infections are also recognized causes. Infective compartment syndrome in the absence of open soft-tissue injuries is rare and requires multidisciplinary management including surgical decompression, debridement, and targeted antimicrobial therapy. We report three cases of upper-limb compartment syndrome secondary to Group A Streptococcush, highlighting diagnostic challenges, surgical management, and reconstructive outcomes.

## Introduction

Acute compartment syndrome (ACS) secondary to infection is rare and is frequently associated with delayed diagnosis and significant morbidity [1]. Group A Streptococcus (GAS) is an especially aggressive pathogen capable of rapid soft-tissue destruction. This case series describes three upper-limb presentations, highlighting diagnostic challenges, surgical management, and reconstructive outcomes.

## Case series

### Case 1

A 22-year-old right-hand-dominant, otherwise healthy female sustained a closed, minimally displaced distal radius fracture of the right wrist following a mechanical fall. She initially presented to the emergency department (ED) and was managed conservatively with cast immobilization. Two days later, she re-presented with increasing pain at the fracture site and pyrexia. Compartment syndrome was excluded by orthopaedics, and she was discharged with analgesia.

**Figure 1:**
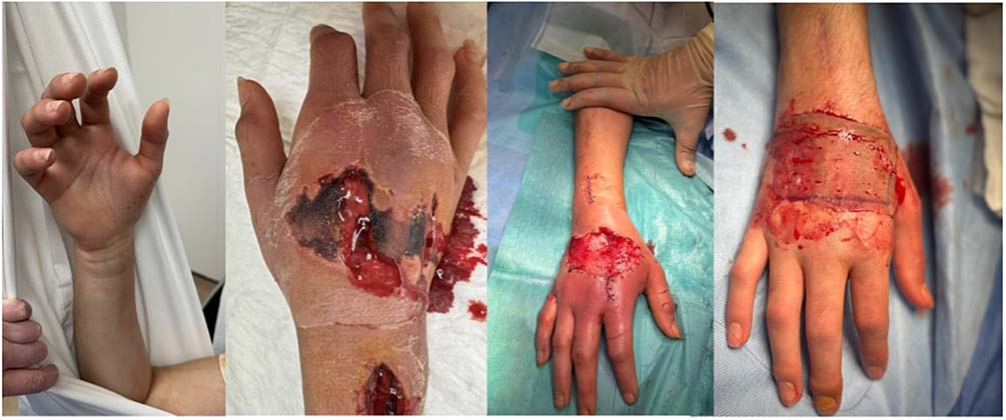
Case 1: Hand on admission, on second washout, following BTM application, and following SSG application.

Twenty-four hours later, she returned with rapidly worsening pain, swelling, and signs of neurovascular compromise (Fig. 1a). There was significant clinical concern for compartment syndrome, and the plastic surgery team was urgently consulted. Examination revealed classical features of compartment syndrome with superimposed severe soft-tissue infection. The hand was diffusely swollen with a tense, mottled dorsum; the fingers were held in a claw posture with no active movement and severe pain on passive extension.

Intravenous antibiotics, fluids, analgesia, and high-arm elevation were commenced immediately. Emergency fasciotomy and debridement were performed. Intraoperatively, frank purulence was identified within the interossei and adductor pollicis compartments with evidence of skin and muscle ischaemia; the thenar and hypothenar compartments were spared. The patient reported a recent upper respiratory tract infection prior to injury. Microbiological analysis of throat swabs, blood cultures, and intraoperative samples all grew GAS. She underwent three further debridement and washout procedures before application of a biodegradable temporizing matrix (BTM) to the dorsum of the hand (Fig. 1b and c). Six weeks later, split-thickness skin grafting (SSG) was performed (Fig. 1d). At 10-month follow-up, she had made a full recovery with restoration of full range of motion following intensive hand therapy.

### Case 2

A 32-year-old right-hand-dominant female with a history of intravenous drug use presented to the ED with a closed left intra-articular distal radius fracture sustained after falling from a second-storey window. She was immobilized in a back slab and discharged. She re-presented the following day with ongoing pain; the slab was removed and reapplied. Forty-eight hours later, she re-attended with severely worsening pain and swelling of the left forearm and hand. She disclosed heroin injection into the left antecubital fossa 24 hours prior.

Examination demonstrated a hot, tense, swollen, and tender forearm with pain on passive extension and reduced sensation in the median and ulnar nerve distributions. There was clinical concern for compartment syndrome and necrotising fasciitis. At fasciotomy, the forearm soft tissues were diffusely swollen with purulent discharge and signs of muscle ischaemia; non-viable muscle was debrided. Post-operatively, she was managed with vancomycin, piperacillin-tazobactam, and metronidazole in accordance with the local necrotising fasciitis protocol. Intraoperative cultures grew GAS.

**Figure 2:**
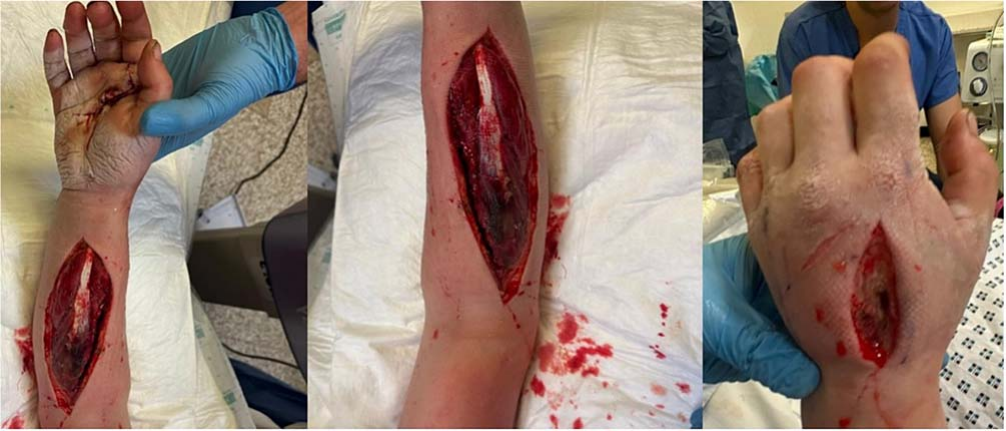
Case 2: Intraoperative photographs at the time of second washout.

At re-exploration 48 hours later, small volumes of pus were identified in both the forearm and hand incisions (Fig. 2). The flexor carpi radialis and flexor digitorum superficialis muscle bellies were non-viable, whilst the interossei and extensor compartments remained viable. Over 6 days, she underwent three additional debridements, progressive wound closure, and vacuum-assisted closure (VAC) therapy. Four weeks after the initial surgery, SSG was performed to the forearm wounds.

### Case 3

**Figure 3:**
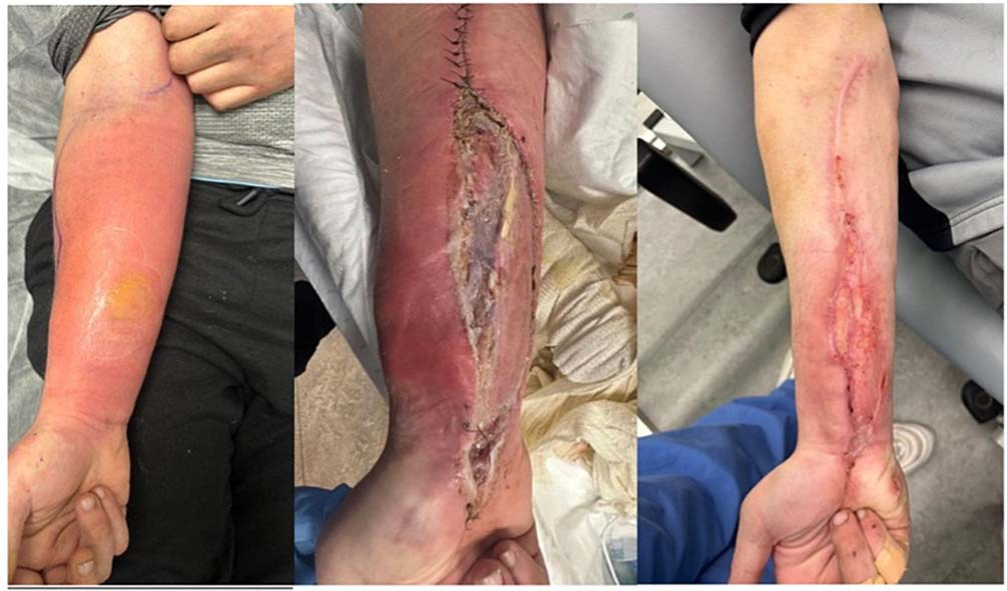
Case 3: Forearm pre-fasciotomy; on second admission following repeat intravenous drug use; on third admission.

A 34-year-old male with a history of intravenous drug use was admitted with extensive oedema and erythema of the right forearm (Fig. 3a). Examination revealed a palpable collection and pain on passive extension. Inflammatory markers were markedly elevated (CRP 169 mg/l; WBC 22.4 × 10^9^/l). Intravenous co-amoxiclav and linezolid were commenced and the forearm was explored under general anaesthesia for suspected compartment syndrome.

Intraoperatively, a subfascial collection containing over 200 ml of pus was evacuated. The brachioradialis, flexor digitorum superficialis, and flexor digitorum profundus muscle bellies were contaminated with compromised viability; non-viable muscle was resected. Cultures grew GAS. A second washout was performed 2 days later with debridement of the brachioradialis, pronator teres, palmaris longus, and flexor digitorum superficialis. By day 5, swelling and pain had improved, and the remaining musculature appeared viable; SSG was applied to the fasciotomy defect. The patient discharged himself against medical advice 48 hours post-grafting.

He was re-admitted 6 days later with cellulitis following further intravenous drug use, which settled with intravenous antibiotics (Fig. 2b). A subsequent re-admission was required for a contralateral upper-limb deep vein thrombosis related to ongoing intravenous drug use (Fig. 2c).

## Discussion

Fractures and soft-tissue injuries are well-established causes of ACS, with some series reporting fracture involvement in up to 70% of cases [2]. In contrast, ACS arising from infection is distinctly uncommon. A review of the literature identified only seven previously reported cases of compartment syndrome attributed to streptococcal infection in the preceding 25 years, underscoring its rarity (Table 1).

**Table 1:** Summary of previously published cases of GAS compartment syndrome.

Author	Year	No.	Age	Limb	Mechanism	Time to theatre	Reconstruction	Outcome/function	LOS
Goyal *et al.* [3]	2025	1	35	Right lower leg	Disseminated streptococcal pharyngitis	2 days	Jacob’s ladder closure; primary closure day 12	Full recovery	18 days
Hernández-Naranjo *et al.* [4]	2024	1	53	Right lower leg	Unknown	N/A	Jacob’s ladder closure followed by SSG	Joint stiffness and hypoaesthesia in external popliteal sciatic nerve distribution	N/A
Samal *et al.* [5]	2024	1	45	Left forearm	Laceration to forearm 15 days prior	5 days	SSG	DASH score: 27% post-rehabilitation	N/A
Toney et al [6]	2016	1	53	Left foot	No trauma; HIV positive	7 days	Secondary intention	Full recovery	10 days
Jamil *et al.* [7]	2011	1	64	Right forearm	Unknown	6 hours	SSG	Functional range of movement at wrist and fingers with mild grip-strength deficit	N/A
Wong *et al.* [8]	2005	1	55	Left leg	Disseminated streptococcal pharyngitis	N/A	VAC dressing	Discharged	14 days
Shah *et al.* [9]	2002	1	37	Right leg	Unknown; diabetic patient	N/A	SSG	Good recovery	N/A

In the absence of a fracture, the diagnosis and timely management of ACS is challenging, often resulting in delayed presentation with risk of irreversible muscle damage. Compared with fracture-associated ACS, non-fracture ACS has been shown to present significantly later to surgical services—some studies citing delays of up to 22 hours on average [1]. Such delays carry important consequences: patients are more likely to undergo late fasciotomy and experience higher rates of muscle necrosis requiring debridement [1]. Consistent with these findings, all three of our patients presented >48 hours after symptom onset and required extensive debridement of necrotic musculature.

Reconstructing the upper limb following infection requires a careful balance between thorough eradication of infection and timely restoration of function, form, and durable soft-tissue coverage. All three cases were ultimately closed with SSG, with the additional use of BTM as an interim measure in one. Coverage choices are guided by the presence of exposed vital structures, the size and depth of the defect, and the patient’s overall condition. Optimal timing requires delaying reconstruction until sepsis is controlled, whilst avoiding postponement that could lead to stiffness or functional decline. La Padula *et al.* reported a mean time to grafting and wound resurfacing in upper-limb necrotising fasciitis of 6 weeks [10].

## Conclusion

This case series emphasizes the exceptional rarity of infective compartment syndrome and the destructive potential of GAS as a causative organism. The complexities involved in reconstructing infected upper-limb tissues—balancing eradication of infection with timely and durable restoration of form and function—further highlight the demanding nature of these presentations. These cases demonstrate the need for prompt diagnosis, decisive surgical management, and a coordinated multidisciplinary approach to optimize limb preservation and long-term functional outcomes.
